# Importance of dialysis specialists in early mortality in elderly hemodialysis patients: a multicenter retrospective cohort study

**DOI:** 10.1038/s41598-024-52170-9

**Published:** 2024-01-22

**Authors:** Yohan Park, Ji Won Lee, Se-Hee Yoon, Sung-Ro Yun, Hyunsuk Kim, Eunjin Bae, Young Youl Hyun, Sungjin Chung, Soon Hyo Kwon, Jang-Hee Cho, Kyung Don Yoo, Woo Yeong Park, In O Sun, Byung Chul Yu, Gang-Jee Ko, Jae Won Yang, Sang Heon Song, Sung Joon Shin, Yu Ah Hong, Won Min Hwang

**Affiliations:** 1 Division of Nephrology, Department of Internal Medicine, Konyang University Hospital, Daejeon, Republic of Korea; 2 Konyang University Myunggok Medical Research Institute, Daejeon, Republic of Korea; 3 Division of Nephrology, Department of Internal Medicine, Hallym University Medical Center, Chuncheon Sacred Heart Hospital, Chuncheon, Republic of Korea; 4 Division of Nephrology, Department of Internal Medicine, Gyeongsang National University Hospital, Jinju, Republic of Korea; 5 Division of Nephrology, Department of Internal Medicine, Kangbuk Samsung Hospital, Seoul, Republic of Korea; 6 Division of Nephrology, Department of Internal Medicine, Yeouido St. Mary’s Hospital, Seoul, Republic of Korea; 7 Division of Nephrology, Department of Internal Medicine, Soonchunhyang University Seoul Hospital, Seoul, Republic of Korea; 8 Division of Nephrology, Department of Internal Medicine, Kyungpook National University Hospital, Daegu, Republic of Korea; 9 Division of Nephrology, Department of Internal Medicine, Ulsan University Hospital, Ulsan, Republic of Korea; 10 Division of Nephrology, Department of Internal Medicine, Keimyung University Dongsan Hospital, Daegu, Republic of Korea; 11 Division of Nephrology, Department of Internal Medicine, Presbyterian Medical Center, Jeonju, Republic of Korea; 12 Division of Nephrology, Department of Internal Medicine, Soonchunhyang University Bucheon Hospital, Bucheon, Republic of Korea; 13 Division of Nephrology, Department of Internal Medicine, Korea University Guro Hospital, Seoul, Republic of Korea; 14 Division of Nephrology, Department of Internal Medicine, Yonsei University Wonju Severance Christian Hospital, Wonju, Republic of Korea; 15 Division of Nephrology, Department of Internal Medicine, Pusan National University Hospital, Busan, Republic of Korea; 16 Division of Nephrology, Department of Internal Medicine, Dongguk University Ilsan Hospital, Goyang, Republic of Korea; 17 Division of Nephrology, Department of Internal Medicine, Daejeon St. Mary’s Hospital, Daejeon, Republic of Korea

**Keywords:** Haemodialysis, Geriatrics

## Abstract

The early mortality rate in elderly patients undergoing hemodialysis is more than twice that in young patients, requiring more specialized healthcare. We investigated whether the number of professional dialysis specialists affected early mortality in elderly patients undergoing hemodialysis. This multicenter retrospective cohort study analyzed data from 1860 patients aged ≥ 70 years who started hemodialysis between January 2010 and December 2017. Study regions included Seoul, Gyeonggi-do, Gangwon-do, Daejeon/Chungcheong-do, Daegu/Gyeongsangbuk-do, and Busan/Ulsan/Gyeongsangnam-do. The number of patients undergoing hemodialysis per dialysis specialist was calculated using registered data from each hemodialysis center. Early mortality was defined as death within 6 months of hemodialysis initiation. Gangwon-do (28.3%) and Seoul (14.5%) showed the highest and lowest early mortality rate, respectively. Similarly, Gangwon-do (64.6) and Seoul (43.9) had the highest and lowest number of patients per dialysis specialist, respectively. Relatively consistent results were observed for the regional rankings of early mortality rate and number of patients per dialysis specialist. Multivariate Cox regression analysis—adjusted for previously known significant risk factors—revealed that the number of patients per dialysis specialist was an independent risk factor for early mortality (hazard ratio: 1.031, *p* < 0.001). This study underscores the growing need for dialysis specialists for elderly hemodialysis patients in Korea.

## Introduction

The incidence of end-stage kidney disease (ESKD) is increasing annually worldwide, with the Republic of Korea (ROK) showing a particularly rapid increase^1,2^. This increase in ESKD incidence is considered to be due to an aging population and increased prevalence of chronic diseases^3^. Notably, the ROK has the lowest fertility rate and the fastest-aging population worldwide^4^. In the ROK, the proportion of patients with ESKD aged ≥ 65 years increased rapidly from 36.0% in 2010 to 51.9% in 2019^1,5^.

Previous studies and reviews have reported that elderly patients undergoing hemodialysis have a 2–4 fold higher mortality rate than their younger counterparts, especially in the early period after hemodialysis initiation^6,7^. The early mortality rate in elderly patients undergoing hemodialysis is more than twice that in their younger counterparts (5.7% for patients aged < 45 years, 19.6% for patients aged 45–64 years, 28.2% for patients aged 65–74 years, and 46.5% for patients aged ≥ 75 years)^7^. Elderly patients undergoing hemodialysis are more prone to comorbidities than younger patients, and its risk increases not only with frailty, malnutrition, and low quality of life but also with age itself; therefore, more detailed and specialized management may be required for elderly patients^8–11^. Thus, careful management by a physician professionally trained in nephrology, or at least in dialysis, is important for the care of elderly patients undergoing hemodialysis^11,12^.

The Korean Society of Nephrology (KSN) is implementing a dialysis specialist system. In addition, a hemodialysis center certification program is being implemented, and the standards for dialysis specialists are reflected in the certification standards^13^. However, the dialysis specialist system is not legally enforced and many hemodialysis centers in the ROK are maintained by doctors who are not dialysis specialists^14^. The performance of hemodialysis by doctors other than dialysis specialists can significantly impact the prognosis of patients, especially that of elderly patients undergoing hemodialysis with a high early mortality rate^15^. Therefore, this study was conducted to examine the importance of dialysis specialists for elderly patients undergoing hemodialysis in the context of early mortality.

## Methods

### Data source and study population

**Figure 1:**
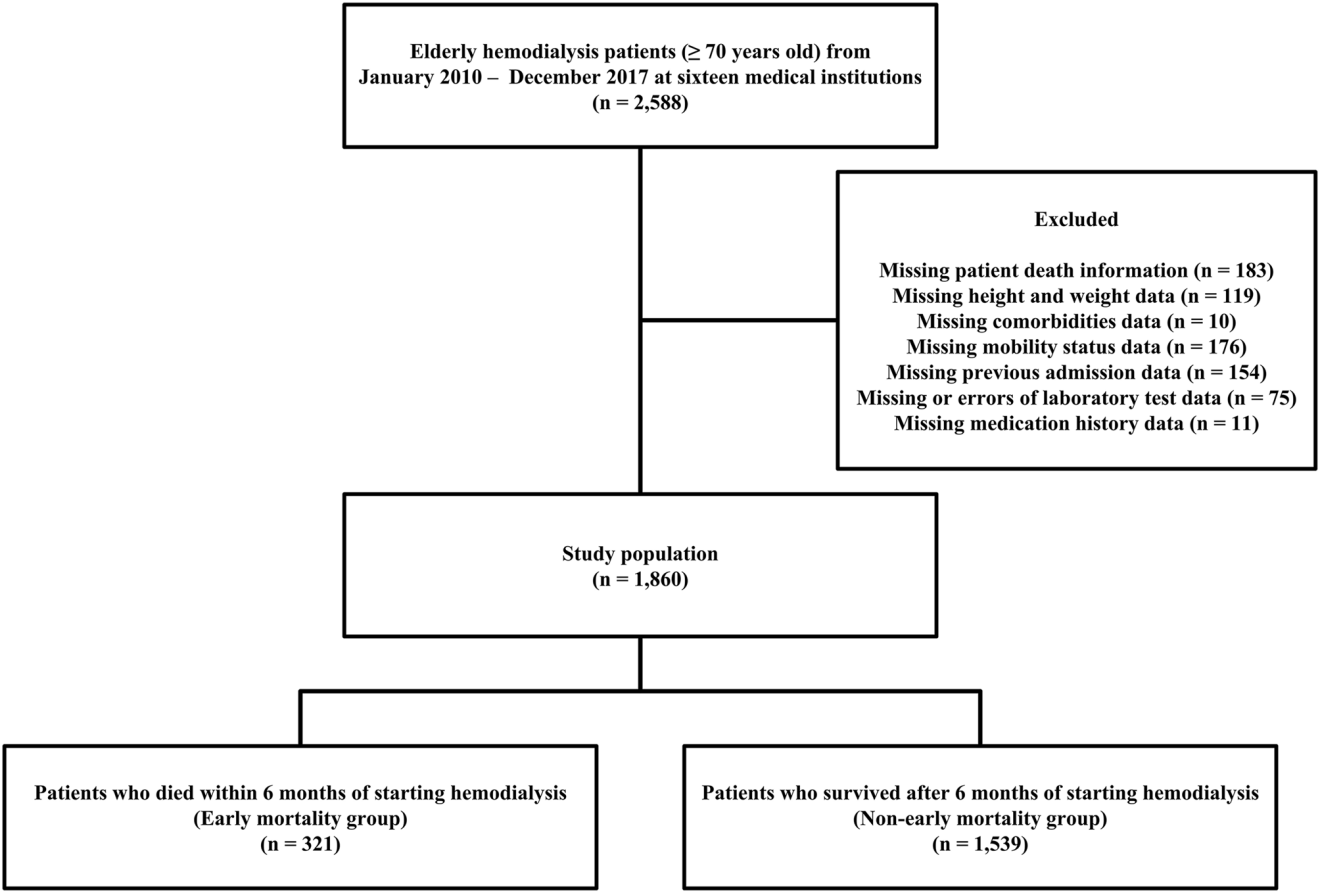
Study design and population. Among 2588 patients aged ≥ 70 years who started hemodialysis at sixteen medical institutions between January 2010 and December 2017, 728 patients with missing or erroneous data were excluded. Finally, 1860 patients were included in the analysis. The early mortality group comprised 321 patients, whereas the non-early mortality group consisted of 1539 patients, corresponding to an early mortality rate of 17.3%.

This multicenter, retrospective, observational cohort study analyzed data from patients aged ≥ 70 years who started hemodialysis between January 2010 and December 2017 at sixteen medical institutions in the ROK belonging to the Korean Society of Geriatric Nephrology. Among the total 2588 patients, 183 with no mortality data, 119 with missing height and weight data, 10 with missing comorbidity data, 176 with missing previous mobility status, 154 with missing previous hospitalization history and nursing home residence data, 75 with errors or missing laboratory test data, and 11 with missing medication history were excluded. Finally, 1860 patients were enrolled in the study. The enrolled patients were classified into the early mortality and non-early mortality groups and their data were compared (Fig. 1). This study adhered to the Declaration of Helsinki and was approved by the Institutional Review Board of Konyang University Hospital (KYUH 2022-12-024). The need to obtain informed patient consent was waived by the Institutional Review Board of Konyang University Hospital (KYUH 2022-10-024) because the patient data were extracted in an anonymized form.

### Calculation of dialysis specialist-related data

Study regions were classified according to the administrative division standards of the ROK as follows: Seoul, Gyeonggi-do, Gangwon-do, Daejeon/Chungcheong-do, Daegu/Gyeongsangbuk-do, and Busan/Ulsan/Gyeongsangnam-do. The number of patients undergoing hemodialysis per dialysis specialist by region was calculated using data from the Korean Renal Data System (KORDS), a dialysis patient registration program implemented by the KSN. The KSN issues a dialysis specialist license to internal medicine specialists who have undergone training in nephrology and have worked at a hemodialysis center for > 2 years after being recommended by two existing dialysis specialists. Physicians with a dialysis license were defined and counted as dialysis specialists. The number of dialysis patients was calculated based on the number of patients whose data were registered at each hemodialysis center. As for transferred patients, the record from the previous hemodialysis center was deleted in the KORDS, and patients newly transferred to a hemodialysis center were regarded as “transferred.” Thus, transferred patients were calculated as the number of patients who were newly transferred to the hemodialysis center.

The fulfillment of recommendations for dialysis specialists at each hemodialysis center was assessed based on the standards of the KSN’s hemodialysis center certification program. The recommended standards for dialysis specialists were ≤ 24, ≤ 26, and ≤ 36 dialysis sessions per dialysis specialist per day in general hospitals, hospitals, and clinics^14^. In the ROK, general hospitals, hospitals, and clinics are defined as hospitals with ≥ 100 beds, 30–99 beds, and ≤ 29 beds, respectively (for more details, see reference^16^). Given that the number of recommended dialysis specialists differs depending on the size of medical institutions, the number of patients per dialysis specialist was calculated at the general hospital, hospital, and clinic levels.

### Clinical parameters and outcomes

Data on demographics, such as height, weight, unplanned dialysis, comorbidities (diabetes mellitus, hypertension, ischemic heart disease, congestive heart failure, cardiac arrhythmia, cerebrovascular disease, peripheral vascular disease, active malignancy, severe behavioral disorder, and liver cirrhosis), mobility status, nursing home residence, hospitalization history within 6 months before dialysis, and laboratory test results were collected at the time of hemodialysis initiation. Patient mortality data were requested from the National Statistical Office of the ROK and collected using the resident registration number. Using the collected data, early mortality prediction scores for elderly patients undergoing hemodialysis were calculated according to the French renal epidemiology and information network (REIN) study, and studies by Santos et al., and Thamer et al. (hereafter referred to as REIN, Santos, and Thamer scores)^17–19^. The REIN score is composed of age, sex, congestive heart failure, peripheral vascular disease, cardiac arrhythmia, active malignancy, severe behavioral disorder, mobility status, and serum albumin (for more details, see reference^17^). The Santos score comprises age, ischemic heart disease, cerebrovascular disease, serum albumin, and prior nephrologist's care, but prior nephrologist's care scores were omitted in the present study as they were not collected (for more details, see reference^18^). The Thamer score encompasses age, serum albumin, mobility status, nursing home residence, malignancy, congestive heart failure, and hospitalization history within 6 months before dialysis (for more details, see reference^19^). The patients were followed up until December 31, 2020. The mortality rate of hemodialysis patients is high within the first 6 months of starting hemodialysis^7,20^. Furthermore, in previous studies on the development of a prediction model for early mortality in elderly hemodialysis patients, death within 6 months of starting hemodialysis was defined as early mortality^17–19,21^. Therefore, in this study, death within 6 months of starting hemodialysis was defined as early mortality and set as the primary outcome.

### Statistical analysis

All continuous variables are expressed as mean ± standard deviation. Student’s t-tests were used to compare continuous variables. Nominal variables are expressed as proportions. The chi-squared test or Fisher’s exact test was used for the comparison of nominal variables, as appropriate. As this study was a retrospective cohort analysis, sample size calculation based on statistical power was not performed, and all patient data during the relevant period were collected from each research institution. Receiver operating characteristic (ROC) curve analysis was performed to set the cut-off value for the number of patients per dialysis specialist, and the value showing the highest sensitivity and specificity in predicting early mortality was set as the cut-off value^22^. Kaplan–Meier survival curve analysis was performed to compare patient survival between the low and high patients per dialysis specialist groups according to the cut-off value, which were compared using the log-rank test. Multivariate Cox proportional hazard regression analysis was performed to identify the risk factors for early mortality in elderly patients undergoing hemodialysis. We conducted 1:2 propensity score matching for factors showing significant differences between the early mortality and non-early mortality groups. Nearest neighbor matching was performed for propensity score matching using the Matchlt package in R 4.1.1 (https://www.R-project.org/). Patients were matched to the closest pair based on the propensity score difference between the two groups. All statistical analyses were performed using SPSS software version 24 (IBM Corporation, Armonk, NY, USA) and Microsoft Excel 2016, with statistical significance set at a *p *value of < 0.05.

## Results

### Comparison of baseline characteristics between early mortality and non-early mortality group

Among 1860 patients, early mortality occurred in 321 patients, corresponding to an early mortality rate of 17.3%. The average follow-up period in the overall cohort was 1193 ± 916 days; the average survival period was 73 ± 52 days in the early mortality group and 1426 ± 835 days in the non-early mortality group. Table 1 compares the baseline characteristics of the early mortality and non-early mortality groups. Age was significantly higher in the early mortality group; furthermore, the early mortality group exhibited significantly lower body mass index (BMI) and lower blood urea nitrogen (BUN), creatinine, albumin, and inorganic phosphorus levels. With respect to comorbidities, the early mortality group showed a significantly higher proportion of cardiac arrhythmia, active malignancy, and severe behavioral disorders but a significantly lower proportion of hypertension. In the early mortality group, the proportion of patients with reduced independent activity, nursing home residence, hospitalization history within 6 months before dialysis, and unplanned dialysis was significantly higher. The REIN, Santos, and Thamer scores were significantly higher in the early mortality group. The number of patients per dialysis specialist and the proportion of centers that did not meet the recommended number of dialysis specialists were significantly higher in the early mortality group.

**Table 1:** Comparison of baseline characteristics between early mortality and non-early mortality groups.

	Early mortality group (n = 321)	Non-early mortality group (n = 1539)	*p* value
Age (years)	79.7 ± 5.9	77.3 ± 5.2	< 0.001
Male (%)	177 (55.1)	867 (56.3)	0.695
Body mass index (kg/m^2^)	22.6 ± 4.0	23.2 ± 3.7	0.007
Comorbidities			
Diabetes mellitus (%)	182 (56.7)	930 (60.4)	0.215
Hypertension (%)	270 (84.1)	1399 (90.9)	< 0.001
Ischemic heart disease (%)	75 (23.4)	351 (22.8)	0.829
Congestive heart failure (%)	65 (20.2)	255 (16.6)	0.112
Cardiac arrhythmia (%)	55 (17.1)	168 (10.9)	0.002
Cerebrovascular disease (%)	59 (18.4)	278 (18.1)	0.894
Peripheral vascular disease (%)	12 (3.7)	98 (6.4)	0.069
Active malignancy (%)	20 (6.2)	44 (2.9)	0.003
Severe behavioral disorder (%)	57 (17.8)	169 (11.0)	0.001
Liver cirrhosis (%)	14 (4.4)	44 (2.9)	0.159
Mobility			< 0.001
Walking without help (%)	134 (41.7)	938 (60.9)	
Needing assistance for transfer (%)	93 (29.0)	394 (25.6)	
Totally dependent for transfer (%)	94 (29.3)	207 (13.5)	
Nursing home residence (%)	52 (16.2)	117 (7.6)	< 0.001
Hospitalization history within 6 months before dialysis (%)	141 (43.9)	527 (34.2)	0.001
Unplanned dialysis (%)	276 (86.0)	1172 (73.2)	< 0.001
Laboratory findings			
Hemoglobin (g/dL)	9.2 ± 1.6	9.2 ± 1.6	0.853
Blood urea nitrogen (mg/dL)	73 ± 37	78 ± 35	0.022
Creatinine (mg/dL)	5.3 ± 2.8	6.6 ± 2.9	< 0.001
Albumin (g/dL)	3.1 ± 0.6	3.4 ± 0.6	< 0.001
Calcium (mg/dL)	8.2 ± 0.9	8.1 ± 1.0	0.115
Inorganic phosphorus (mg/dL)	4.7 ± 1.9	5.0 ± 2.0	0.044
REIN score	8.6 ± 5.0	5.5 ± 4.4	< 0.001
Santos score^a^	3.6 ± 1.7	2.8 ± 1.7	< 0.001
Thamer score	3.5 ± 1.3	2.6 ± 1.2	< 0.001
Number of patients per dialysis specialist	56.3 ± 7.1	54.9 ± 7.0	0.002
Proportions of centers that did not meet the recommended number of dialysis specialists (%)	50.2 ± 11.4	48.3 ± 11.6	0.007

Continuous and categorical variables are presented as mean ± standard deviation and as number (%), respectively.

REIN, renal epidemiology and information network cohort.

^a^The Santos score consisted of age, ischemic heart disease, cerebrovascular disease, albumin, and prior nephrologist’s care; however, the prior nephrologist’s care score was omitted as this was not collected in the study.

### Comparison of early mortality and number of dialysis specialists by region in the ROK

**Figure 2:**
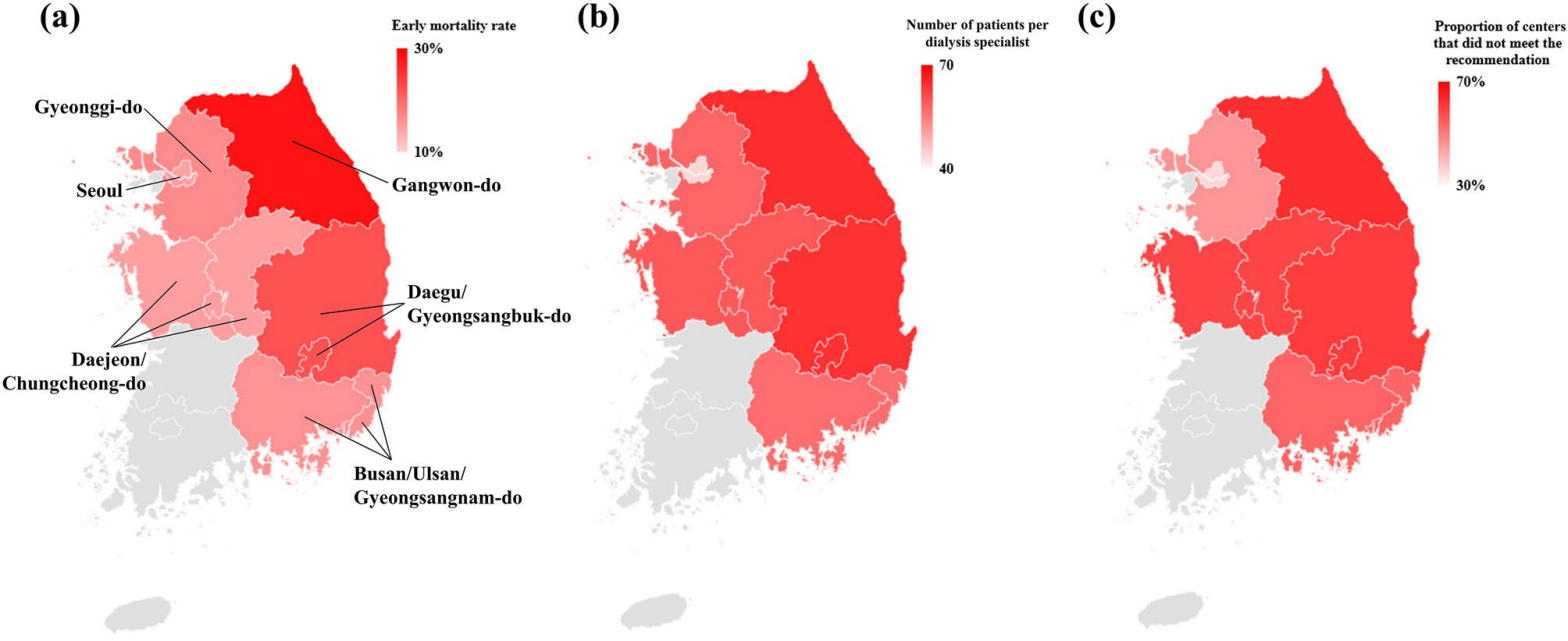
**(a)** Early mortality, **(b)** number of patients per dialysis specialist, and **(c)** proportion of centers that did not meet the recommended number of dialysis specialists according to the regions of the Republic of Korea. The early mortality rate was the highest in Gangwon-do, followed by Daegu/Gyeongsangbuk-do, Gyeonggi-do, Busan/Ulsan/Gyeongsangnam-do, Daejeon/Chungcheong-do, and Seoul. Gangwon-do had the highest number of patients per dialysis specialist, followed by Daegu/Gyeongsangbuk-do, Daejeon/Chungcheong-do, Gyeonggi-do, Busan/Ulsan/Gyeongsangnam-do, and Seoul. Additionally, Gangwon-do had the highest proportion of centers that did not meet the recommended number of dialysis specialists, followed by Daegu/Gyeongsangbuk-do, Daejeon/Chungcheong-do, Busan/Ulsan/Gyeongsangnam-do, Gyeonggi-do, and Seoul. The regional rankings for the early mortality rate, number of patients per dialysis specialist, and proportion of centers that did not meet the recommended number of dialysis specialists were relatively consistent (created with Bing, Geonames Tomtom, Microsoft, produced by Microsoft Excel 2016).

The early mortality rate was the highest in Gangwon-do (28.3%), followed by Daegu/Gyeongsangbuk-do (22.2%), and the lowest in Seoul (14.5%, Fig. 2a). The number of patients per dialysis specialist was also the highest in Gangwon-do (64.6), followed by Daegu/Gyeongsangbuk-do (63.9), and the lowest in Seoul (43.9, Fig. 2b). Gangwon-do had the highest proportion of centers that did not meet the recommended number of dialysis specialists (62.5%), followed by Daegu/Gyeongsangbuk-do (60.2%); conversely, Seoul had the lowest proportion of such centers (30.3%, Fig. 2c). Relatively consistent results were observed for the regional rankings of the early mortality rate, number of patients per dialysis specialist, and proportion of centers that did not meet the recommended number of dialysis specialists (Fig. 2). Overall, 57,167 patients were registered in the KORDS database, of whom 25,696 (44.9%) were in clinics, 7963 (13.9%) in hospitals, and 23,508 (41.1%) in general hospitals (Supplementary Table S1 online). Except for Busan/Ulsan/Gyeongsangnam-do, the number of patients per dialysis specialist in all regions was lower in general hospitals than at hospitals and clinics. Nevertheless, the proportion of centers that did not meet the recommended number of dialysis specialists varied by region. Notably, Busan/Ulsan/Gyeongsangnam-do showed a low proportion of unmet hemodialysis centers with dialysis specialists at the clinic level (39.3% in clinics vs. 60.4% in hospitals and 63.6% in general hospitals). In contrast, Gangwon-do exhibited a high proportion of unmet hemodialysis centers with dialysis specialists at the general hospital level (72.7% at the general hospitals vs. 58.3% at the clinics and 55.6% at the hospitals). The median survival duration was the lowest in Gangwon-do (Table 2).

**Table 2:** Comparison of early mortality and dialysis specialists in the Republic of Korea according to region.

	Seoul (n = 470)	Gyeonggi-do (n = 251)	Gangwon-do (n = 180)	Daejeon/Chungcheong-do (n = 196)	Daegu/Gyeongsangbuk-do (n = 135)	Busan/Ulsan/Gyeongsangnam-do (n = 628)
Early mortality rate (death ≤ 6 months from starting hemodialysis) (%)	68 (14.5)	42 (16.7)	51 (28.3)	29 (14.8)	30 (22.2)	101 (16.1)
Number of patients per dialysis specialist	43.9	57.7	64.6	59.2	63.9	56.7
Clinic level	58.3	61.1	80.4	80.4	73.3	56.1
Hospital level	28.8	66.0	69.8	79.4	61.5	58.0
General hospital level	34.1	51.9	55.0	41.7	57.8	56.5
Proportions of centers that did not meet the recommended number of dialysis specialists (n/n, %)	63/208 (30.3)	104/226 (46.0)	20/32 (62.5)	63/107 (58.9)	59/98 (60.2)	88/164 (53.7)
Clinic level	37/122 (30.3)	51/122 (41.8)	7/12 (58.3)	28/48 (58.3)	29/47 (61.7)	24/61 (39.3)
Hospital level	12/37 (32.4)	34/52 (65.4)	5/9 (55.6)	21/26 (80.8)	12/22 (54.5)	29/48 (60.4)
General hospital level	14/49 (28.6)	19/52 (36.5)	8/11 (72.7)	14/33 (42.4)	18/29 (62.1)	35/55 (63.6)
Median survival duration (days, IQR)	1152 (420–1790)	1081 (301–1826)	820 (132–1492)	1091 (450–1741)	1081 (214–1805)	1234 (437–1753)

Continuous and categorical variables are presented as median (interquartile range) and proportions, respectively.

*IQR* interquartile range.

### Kaplan–Meier survival curve analysis of the low and high patients per dialysis specialist groups

**Figure 3:**
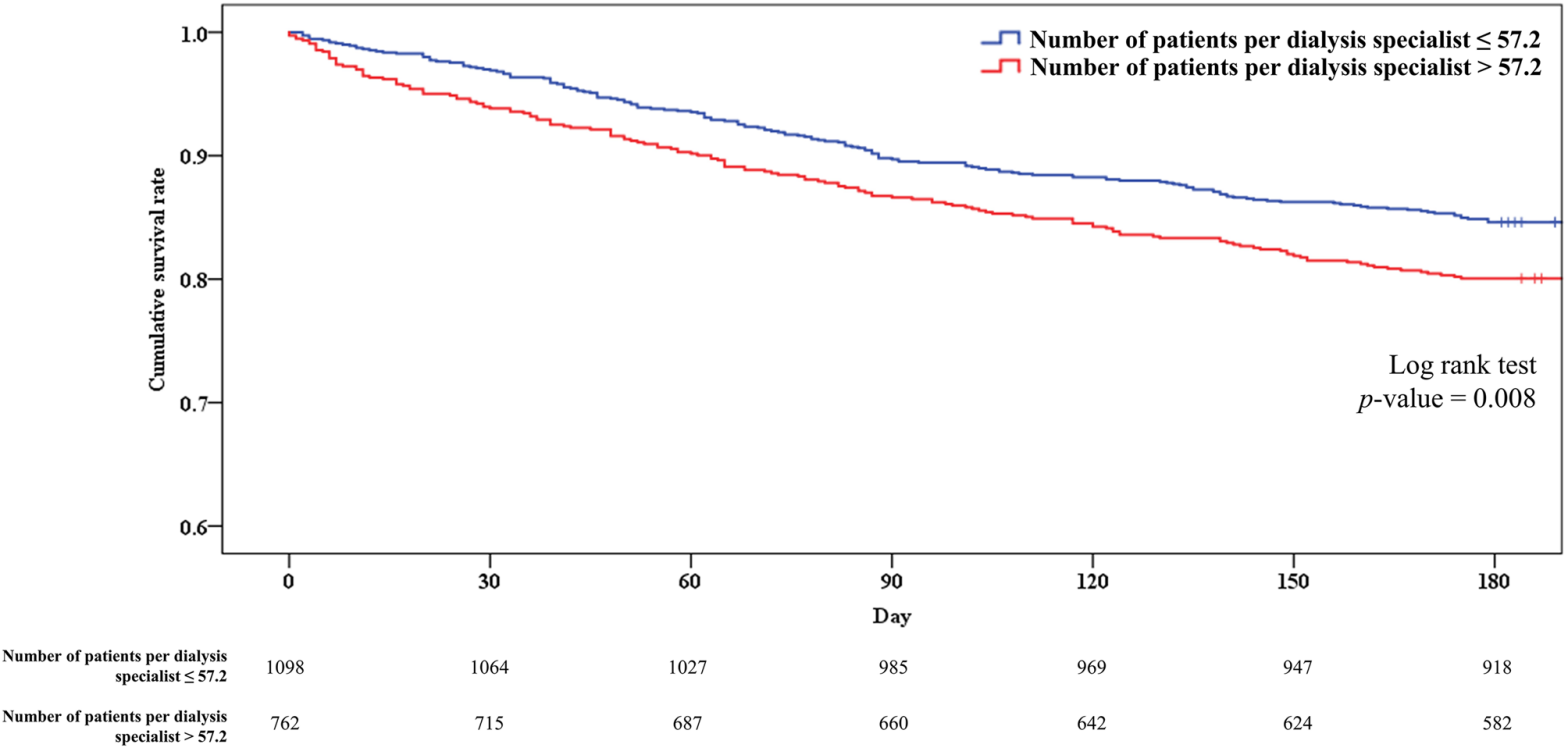
Kaplan–Meier survival curve analysis of the low and high patients per dialysis specialist groups. There was a clear difference in survival between the two groups. The early mortality rate was 16.4% in the low group and 23.6% in the high group.

The cut-off value for the number of patients per dialysis specialist set by ROC curve analysis was 57.2. Based on this, the patients were classified into the low and high patients per dialysis specialist groups, and Kaplan–Meier survival curve analysis was performed. There was a clear difference in survival between the two groups with a log-rank test *p* value of 0.008 (Fig. 3).

### Multivariate Cox proportional hazards regression model analysis for early mortality

Table 3 shows the results of the multivariate Cox proportional hazards regression model analysis conducted to identify the significant factors affecting early mortality. Baseline characteristics showing significant differences between the early mortality and non-early mortality groups (age, BMI, hypertension, cardiac arrhythmia, active malignancy, severe behavioral disorder, mobility status, nursing home residence, hospitalization history within 6 months before dialysis, unplanned dialysis, BUN, creatinine, albumin, and inorganic phosphorus levels), factors that were observed to be important in previous prediction models for early mortality in elderly patients undergoing hemodialysis (sex, congestive heart failure, and peripheral vascular disease), and the number of patients per dialysis specialist were included in the analysis.

**Table 3:** Multivariate Cox proportional hazards regression model analysis for early mortality.

	Univariate HR (95% CI)	Multivariate HR (95% CI)
Age (per 1 years)	1.073 (1.053–1.093)^a^	1.054 (1.034–1.074)^a^
Female (Ref. male)	1.151 (0.844–1.310)	–
Body mass index (per 1 kg/m^2^)	0.958 (0.930–0.988)^a^	–
Hypertension (Ref. No)	0.564 (0.418–0.760)^a^	–
Congestive heart failure (Ref. No)	1.252 (0.954–1.644)	–
Cardiac arrhythmia (Ref. No)	1.614 (1.207–2.158)^a^	-
Peripheral vascular disease (Ref. No)	0.598 (0.336–1.064)	0.553 (0.302–1.013)
Active malignancy (Ref. No)	2.061 (1.311–3.241)^a^	2.066 (1.302–3.277)^a^
Severe behavioral disorder (Ref. No)	1.651 (1.240–2.198)^a^	–
Mobility		
Walking without help	Ref	Ref
Needing assistance for transfer	1.584 (1.216–2.064)^a^	1.211 (0.917–1.599)
Totally dependent for transfer	2.875 (2.209–3.744)^a^	1.822 (1.367–2.427)^a^
Nursing home residence (Ref. No)	2.132 (1.584–2.869)^a^	1.369 (0.997–1.878)
Hospitalization history within 6 months before dialysis (Ref. No)	1.466 (1.176–1.828)^a^	–
Unplanned dialysis (Ref. planned)	2.110 (1.540–2.892)^a^	1.546 (1.117–2.138)^a^
Blood urea nitrogen (per 1 mg/dL)	0.996 (0.993–0.999)^a^	–
Creatinine (per 1 mg/dL)	0.828 (0.788–0.870)^a^	0.900 (0.857–0.944)^a^
Albumin (per 1 g/dL)	0.446 (0.376–0.529)^a^	0.536 (0.444–0.648)^a^
Inorganic phosphorus (per 1 mg/dL)	0.927 (0.864–0.995)^a^	–
Number of patients per dialysis specialist (per 1 patient)	1.028 (1.011–1.045)^a^	1.031 (1.015–1.049)^a^

The multivariate model was adjusted for known significant factors and those showing statistical differences between the early mortality and non-early mortality groups. The following parameters were used: age, sex, body mass index, hypertension, congestive heart failure, cardiac arrhythmia, peripheral vascular disease, active malignancy, severe behavioral disorder, mobility status, nursing home residence, hospitalization history within 6 months before dialysis, unplanned dialysis, blood urea nitrogen, creatinine, albumin, inorganic phosphorus levels, and number of dialysis specialists. After excluding patients with missing values, 1805 (97.0%) participants were included in the multivariate model.

*CI* confidence interval, *HR* hazard ratio, *Ref.* reference.

^a^*p* value < 0.05.

Age, active malignancy, mobility status (totally dependent on transfers), unplanned dialysis, creatinine levels, albumin levels, and number of patients per dialysis specialist were identified as significant factors influencing early mortality in the multivariate model. The risk of early mortality increased by 3.1% when the number of patients per dialysis specialist increased by one.

Table 4 presents the results of the Cox proportional hazards regression model analysis conducted to examine the effect of the number of patients per dialysis specialist at the clinic, hospital, and general hospital levels. The multivariate model included factors reported by previous studies to be important and showing significant differences in baseline characteristics (i.e., the same as the factors included in Table 3; Supplementary Tables S2, S3, and S4 online). The multivariate hazard ratio for the number of patients per dialysis specialist was the highest at the general hospital level (1.025), as compared with the clinic level (1.013) and hospital level (1.009).

**Table 4:** Univariate and multivariate hazard ratios for early mortality in relation to the number of patients per dialysis specialist at the clinic, hospital, and general hospital levels.

Number of patients per dialysis specialist by size of medical institutions	Univariate HR (95% CI)	Multivariate HR (95% CI)
Number of patients per dialysis specialist in clinic level^a^	1.017 (1.006–1.028)	1.013 (1.001–1.024)
Number of patients per dialysis specialist in hospital level^b^	1.007 (1.000–1.013)	1.009 (1.002–1.015)
Number of patients per dialysis specialist in general hospital level^c^	1.014 (1.002–1.026)	1.025 (1.012–1.039)

The multivariate model was adjusted for known significant factors and those showing statistical differences between the early mand non-early mortality groups (age, sex, body mass index, hypertension, congestive heart failure, cardiac arrhythmia, peripheral vascular disease, active malignancy, severe behavioral disorder, mobility status, nursing home residence, hospitalization history within 6 months before dialysis, unplanned dialysis, blood urea nitrogen, creatinine, albumin, inorganic phosphorus levels).

*HR* hazard ratio, *CI* confidence interval.

^a^Number of patients per dialysis specialist at the clinic level was used.

^b^Number of patients per dialysis specialist at the hospital level was used.

^c^Number of patients per dialysis specialist at the general hospital level was used.

### Multivariate Cox proportional hazards regression model analysis for early mortality in the propensity score matched cohort

Table 5 shows the results of the multivariate Cox proportional hazards regression model analysis conducted to identify the significant factors affecting early mortality in the propensity score matched cohort. Baseline characteristics showing significant differences between the early mortality and non-early mortality groups in the propensity score matched cohort (hypertension, cardiac arrhythmia, peripheral vascular disease, active malignancy, mobility status, nursing home residence, hospitalization history within 6 months before dialysis, unplanned dialysis, calcium levels), and the number of patients per dialysis specialist were included in the analysis (Supplementary Table S5). The number of patients per dialysis specialist remained an independent risk factor for early mortality.

**Table 5:** Multivariate Cox proportional hazards regression model analysis for early mortality in the propensity score matched cohort.

	Univariate HR (95% CI)	Multivariate HR (95% CI)
Hypertension (Ref. No)	0.723 (0.536–0.975)^a^	–
Cardiac arrhythmia (Ref. No)	1.318 (0.986–1.762)	–
Peripheral vascular disease (Ref. No)	0.628 (0.353–1.117)	0.617 (0.346–1.102)
Active malignancy (Ref. No)	1.658 (1.054–2.607)^a^	1.789 (1.132–2.827)^a^
Mobility		
Walking without help	Ref	Ref
Needing assistance for transfer	1.119 (0.859–1.458)	1.072 (0.814–1.411)
Totally dependent for transfer	1.899 (1.459–2.473)^a^	1.699 (1.277–2.261)^a^
Nursing home residence (Ref. No)	1.529 (1.136–2.058)^a^	1.364 (0.995–1.870)
Hospitalization history within 6 months before dialysis (Ref. No)	1.247 (1.000–1.554)	–
Unplanned dialysis (Ref. planned)	1.445 (1.055–1.980)^a^	1.430 (1.035–1.975)^a^
Calcium (per 1 mg/dL)	1.159 (1.028–1.307)^a^	1.144 (1.016–1.287)^a^
Number of patients per dialysis specialist (per 1 patient)	1.030 (1.013–1.047)^a^	1.031 (1.015–1.048)^a^

The multivariate model was adjusted for known significant factors and those showing statistical differences between the early mortality and non-early mortality groups in the propensity score matched cohort. The following parameters were used: hypertension, cardiac arrhythmia, peripheral vascular disease, active malignancy, mobility status, nursing home residence, hospitalization history within 6 months before dialysis, unplanned dialysis, calcium levels, and number of dialysis specialists. After excluding patients with missing values, 963 (97.8%) participants were included in the multivariate model.

*CI* confidence interval, *HR* hazard ratio, *Ref.* reference.

^a^*p* value < 0.05.

## Discussion

Herein, we observed that the number of patients per dialysis specialist was significantly higher in the early mortality group than in the non-early mortality group. Similarly, the proportion of centers that did not meet the recommended number of dialysis specialists was higher in the early mortality group. Notably, the number of patients per dialysis specialist was an independent risk factor for early mortality in elderly patients undergoing hemodialysis, even in the propensity score matched cohort.

Regarding baseline characteristics, the early mortality group had significantly lower BMI and albumin levels than the non-early mortality group. Additionally, the proportions of cardiac arrhythmia, active malignancy, severe behavioral disorder, total dependence on transfers, hospitalization history within 6 months before dialysis, and unplanned dialysis were significantly higher in the early mortality group than in the non-early mortality group. These results are consistent with those of many previous studies on early mortality in elderly patients^18,19,21,23,24^. BUN, creatinine, and inorganic phosphorus levels were significantly lower in the early mortality group than in the non-early mortality group, which may be a result of the decreased nutritional status in the early mortality group^25–27^. In addition, the prevalence of hypertension was significantly lower in the early mortality group; however, the reason for this was unclear. It is possible that the use of renin–angiotensin–aldosterone system inhibitor (RAASI) reflects a reduction in mortality, and the low RAASI use in the early mortality group (36.1% in the early mortality group vs. 51.6% in the non-early mortality group) was associated with a low prevalence of hypertension during the retrospective data collection process^28–30^.

Several studies have been conducted to develop prediction models for early mortality in elderly patients undergoing hemodialysis. Although there are slight differences between the models, they commonly include patient age, functional status, hospitalization history, unplanned dialysis, and nutritional status as major factors^17–19,21^. Previous studies have focused on determining whether conservative management should be performed in patients with a high probability of early mortality as they may not benefit sufficiently from hemodialysis^17^. However, while it is important to determine the appropriateness of conservative management in elderly patients undergoing hemodialysis at high risk of early mortality, efforts to reduce early mortality in these patients are also important.

Previous studies have analyzed the early mortality in elderly patients undergoing hemodialysis, focusing only on patient factors. However, early mortality in elderly patients undergoing hemodialysis may also be affected by higher-quality medical care, such as more specialized and meticulous management by dialysis specialists. Therefore, this study focused on physician factors rather than patient factors. In addition to dialysis, the management of patients undergoing hemodialysis also requires integrated management such as anemia, mineral bone disorder, blood pressure control, nutritional status improvement, dialysis adequacy, and vascular access management^31–33^. For meticulous management, the physician in charge of hemodialysis should be trained through a specialized educational program.

The mortality rate in elderly patients undergoing hemodialysis was the highest in Gangwon-do and lowest in Seoul. Similarly, the number of patients per dialysis specialist and the proportion of centers that did not meet the KSN recommendations were the highest in Gangwon-do and lowest in Seoul. When analyzed by region, the rankings for the early mortality rate and number of patients per dialysis specialist were relatively consistent. In addition, based on a cut-off of 57.2 patients per dialysis specialist, there was a clear difference in the Kaplan–Meier survival curve between the low and high patients per dialysis specialist groups. The early mortality rate was 16.4% in the low group and 23.6% in the high group. Furthermore, after adjustment for various factors influencing early mortality in elderly patients undergoing hemodialysis, the number of patients per dialysis specialist was observed to be an independent risk factor for early mortality. In the multivariate Cox regression analysis that included the REIN score previously developed for predicting early mortality in elderly patients undergoing hemodialysis, the number of patients per dialysis specialist was also identified as an independent risk factor (hazard ratio: 1.034, *p* < 0.001). In the propensity score matched cohort analysis conducted to minimize selection bias, the number of patients per dialysis specialist remained an independent risk factor. This suggests that physician factors, represented by dialysis specialists, are important in patient prognosis even after correcting for important patient factors in the early mortality of elderly patients undergoing hemodialysis.

After hemodialysis initiation, patients are usually transferred to the community; in particular, when their risk of early mortality is deemed to be high, they are often transferred to nursing hospitals. Therefore, we additionally analyzed the number of patients per dialysis specialist and centers that did not meet the recommended number of dialysis specialists in clinics, hospitals, and general hospitals. In most regions, there were more dialysis specialists in general hospitals than in hospitals or clinics. In Gangwon-do, the number of patients per dialysis specialist was lower at the general hospital level than at the hospital and clinic levels; nonetheless, the proportion of centers that did not meet the recommended number of dialysis specialists was abnormally high at the general hospital level, which might be attributable to an imbalance in the number of dialysis specialists among general hospitals in this region. General hospitals managing 41.1% of all patients undergoing hemodialysis and the number of patients per dialysis specialist in general hospitals showed the greatest impact on early mortality (Table 4). In other words, both the difference in the supply of dialysis specialists by region and lack of dialysis specialists in general hospital-sized medical institutions have some impact on early mortality. In particular, looking at Gangwon-do, which has the highest early mortality rate, the imbalance in the supply of dialysis specialists among general hospitals in the region can be assumed to be also important.

This study has several limitations. First, it did not include data from Jeolla-do and Jeju-do regions. This is because, at the time of the study, medical institutions in the Jeolla-do and Jeju-do regions were not included in the Korean Society of Geriatric Nephrology; as such, it was not possible to collect data from these regions. More objective results can be obtained by analyzing data from all regions of the ROK.

Second, the number of patients per dialysis specialist was calculated using data from the KORDS database; however, since the data in the KORDS database is voluntarily deposited by hemodialysis centers, accurate reflection of each region using the data is limited. Moreover, hemodialysis centers that deposit data in the KORDS database are likely to have dialysis specialists or physicians interested in the KSN. In other words, it is possible that the number of dialysis specialists in non-metropolitan areas was overestimated because the database contains data deposited by physicians who are interested in the KSN, and in non-metropolitan areas, the number of dialysis specialists is expected to be small. Nevertheless, the results of this study showed that there were fewer dialysis specialists in non-metropolitan areas, which had a significant effect on early mortality in elderly patients undergoing hemodialysis.

Third, the degree of medical access in each region could not be analyzed. Professional care provided by dialysis specialists is important in the context of early mortality in elderly patients undergoing hemodialysis; however, easy-to-access hemodialysis centers are also important. The number of hemodialysis centers registered in the KORDS data was significantly lower for Gangwon-do than for Seoul and Gyeonggi-do. Accordingly, differences in access to medical services among regions might also possibly have an impact on early mortality.

However, our study has strength in that this multicenter study recruited a relatively large number of elderly patients undergoing hemodialysis distributed nationwide. In the analysis using the previously described REIN, Thamer and Santos scores, the results were relatively consistent with the validation cohort analysis results of previous studies; thus, our cohort can be considered reliable for elderly patients undergoing hemodialysis (Supplementary Table S6 online). In addition, to our knowledge, this is the first study to show that the quality of medical services, represented by the number of patients per dialysis specialist, has a significant effect on early mortality in elderly patients undergoing hemodialysis.

In conclusion, the number of patients per dialysis specialist is an important factor for early mortality in elderly patients undergoing hemodialysis. Additionally, the imbalance between regions with dialysis specialists and among medical institutions can affect early mortality in elderly patients undergoing hemodialysis. This study demonstrates the importance of professionally trained dialysis specialists for the rapidly increasing number of elderly patients undergoing hemodialysis in the super-aging society of the ROK. Active introduction of the dialysis specialist system and active training of dialysis specialists are crucial for improving the prognosis of elderly patients undergoing hemodialysis in the future.

### Supplementary Information

Supplementary Information.

## Data Availability

The datasets generated and analyzed during the present study are available from the corresponding author on reasonable request. The data are not publicly available due to privacy or ethical restrictions.
