# Author Correction: Importance of dialysis specialists in early mortality in elderly hemodialysis patients: a multicenter retrospective cohort study

**DOI:** 10.1038/s41598-024-55894-w

**Published:** 2024-03-04

**Authors:** Yohan Park, Ji Won Lee, Se-Hee Yoon, Sung-Ro Yun, Hyunsuk Kim, Eunjin Bae, Young Youl Hyun, Sungjin Chung, Soon Hyo Kwon, Jang-Hee Cho, Kyung Don Yoo, Woo Yeong Park, In O Sun, Byung Chul Yu, Gang-Jee Ko, Jae Won Yang, Sang Heon Song, Sung Joon Shin, Yu Ah Hong, Won Min Hwang

**Affiliations:** 1 Division of Nephrology, Department of Internal Medicine, Konyang University Hospital, Daejeon, Republic of Korea; 2 Konyang University Myunggok Medical Research Institute, Daejeon, Republic of Korea; 3 Division of Nephrology, Department of Internal Medicine, Hallym University Medical Center, Chuncheon Sacred Heart Hospital, Chuncheon, Republic of Korea; 4 Division of Nephrology, Department of Internal Medicine, Gyeongsang National University Hospital, Jinju, Republic of Korea; 5 Division of Nephrology, Department of Internal Medicine, Kangbuk Samsung Hospital, Seoul, Republic of Korea; 6 Division of Nephrology, Department of Internal Medicine, Yeouido St. Mary’s Hospital, Seoul, Republic of Korea; 7 Division of Nephrology, Department of Internal Medicine, Soonchunhyang University Seoul Hospital, Seoul, Republic of Korea; 8 Division of Nephrology, Department of Internal Medicine, Kyungpook National University Hospital, Daegu, Republic of Korea; 9 Division of Nephrology, Department of Internal Medicine, Ulsan University Hospital, Ulsan, Republic of Korea; 10 Division of Nephrology, Department of Internal Medicine, Keimyung University Dongsan Hospital, Daegu, Republic of Korea; 11 Division of Nephrology, Department of Internal Medicine, Presbyterian Medical Center, Jeonju, Republic of Korea; 12 Division of Nephrology, Department of Internal Medicine, Soonchunhyang University Bucheon Hospital, Bucheon, Republic of Korea; 13 Division of Nephrology, Department of Internal Medicine, Korea University Guro Hospital, Seoul, Republic of Korea; 14 Division of Nephrology, Department of Internal Medicine, Yonsei University Wonju Severance Christian Hospital, Wonju, Republic of Korea; 15 Division of Nephrology, Department of Internal Medicine, Pusan National University Hospital, Busan, Republic of Korea; 16 Division of Nephrology, Department of Internal Medicine, Dongguk University Ilsan Hospital, Goyang, Republic of Korea; 17 Division of Nephrology, Department of Internal Medicine, Daejeon St. Mary’s Hospital, Daejeon, Republic of Korea

Correction to: *Scientific Reports* 10.1038/s41598-024-52170-9, published online 22 January 2024

The Funding section in the original version of this Article was incomplete.

"This study was supported by the Konyang University Myunggok Research Fund (2021-02) and the National Research Foundation of Korea (NRF) grant funded by the Korean government (MSIT) (No. RS-2022-00166592). This manuscript is a researcher-led paper with no specific participation by funders.”

now reads:

"This study was supported by the Konyang University Myunggok Research Fund (2021-02), the National Research Foundation of Korea (NRF) grant funded by the Korean government (MSIT) (No. RS-2022-00166592), and the Cooperative Research Grant 2019 from the Korean Society of Nephrology. This manuscript is a researcher-led paper with no specific participation by funders.”

The original Article has been corrected.

