# Supplementary material for: Importance of dialysis specialists in early mortality in elderly hemodialysis patients: a multicenter retrospective cohort study
Source: Sci Rep. 2024 Jan 22;14:1927. doi: 10.1038/s41598-024-52170-9 (PMC10803298; doi:10.1038/s41598-024-52170-9)
Supplement: Supplementary file 1 — Supplementary Information. [file 41598_2024_52170_MOESM1_ESM.docx]

Supplementary Material

**Importance of Dialysis Specialists in Early Mortality in Elderly Hemodialysis Patients: A Multicenter Retrospective Cohort Study**

Yohan Park^1,2^, Ji Won Lee^1^, Se-Hee Yoon^1^, Sung-Ro Yun^1^, Hyunsuk Kim^3^, Eunjin Bae^4^, Young Youl Hyun^5^, Sungjin Chung^6^, Soon Hyo Kwon^7^, Jang-Hee Cho^8^, Kyung Don Yoo^9^, Woo Yeong Park^10^, In O Sun^11^, Byung Chul Yu^12^, Gang-Jee Ko^13^, Jae Won Yang^14^, Sang Heon Song^15^, Sung Joon Shin^16^, Yu Ah Hong^17^, and Won Min Hwang^1*^

*** Correspondence:** Won Min Hwang, MD, PhD: [hwangwm@kyuh.ac.kr](mailto:hwangwm@kyuh.ac.kr)

# Supplementary Tables

**Supplementary Table S1. Number of hemodialysis patients and dialysis specialists in Republic of Korea by region based on KORDS data**

|  | Total | Seoul | Gyeonggi-do | Gangwon-do | Daejeon/ Chungcheong-do | Daegu/ Gyeongsangbuk-do | Busan/ Ulsan/ Gyeongsangnam-do |
| --- | --- | --- | --- | --- | --- | --- | --- |
| **Patients (n, %)** | 57167 (100) | 13926 (100) | 15691 (100) | 2133 (100) | 6753 (100) | 7545 (100) | 11119 (100) |
| Clinic level (n, %) | 25696 (44.9) | 7983 (57.3) | 7701 (49.1) | 724 (33.9) | 3054 (45.2) | 3151 (41.8) | 3083 (27.7) |
| Hospital level (n, %) | 7963 (13.9) | 1038 (7.5) | 1915 (12.2) | 419 (19.6) | 1111 (16.5) | 984 (13.0) | 2496 (22.4) |
| General hospital level (n, %) | 23508 (41.1) | 4905 (35.2) | 6075 (38.7) | 990 (46.4) | 2588 (38.3) | 3410 (45.2) | 5540 (49.8) |
| **Dialysis specialists (n, %)** | 1050 (100) | 317 (100) | 272 (100) | 33 (100) | 114 (100) | 118 (100) | 196 (100) |
| Clinic level (n, %) | 408 (38.9) | 137 (43.2) | 126 (46.3) | 9 (27.3) | 38 (33.3) | 43 (36.4) | 55 (28.1) |
| Hospital level (n, %) | 144 (13.7) | 36 (11.4) | 29 (10.7) | 6 (18.2) | 14 (12.3) | 16 (13.6) | 43 (21.9) |
| General hospital level (n, %) | 498 (47.4) | 144 (45.4) | 117 (43.0) | 18 (54.5) | 62 (54.4) | 59 (50.0) | 98 (50.0) |

Categorical variables are shown as proportions.
KORDS, Korean Renal Data System

**Supplementary Table S2. Multivariate Cox proportional hazards regression model analysis for early mortality of the number of patients per dialysis specialist at clinic level**

|  | **Multivariate HR (95% CI)** |
| --- | --- |
| **Age (per 1 years)** | 1.054 (1.034–1.075) ^a)^ |
| **Female (Ref. male)** | - |
| **Body mass index (per 1 kg/m^2^)** | - |
| **Hypertension (Ref. No)** | - |
| **Congestive heart failure (Ref. No)** | - |
| **Cardiac arrhythmia (Ref. No)** | - |
| **Peripheral vascular disease (Ref. No)** | 0.578 (0.315–1.061) |
| **Active malignancy (Ref. No)** | 1.822 (1.152–2.880) ^a)^ |
| **Severe behavioral disorder (Ref. No)** | - |
| **Mobility** |  |
| Walking without help | Ref. |
| Needing assistance for transfers | 1.134 (0.860–1.497) |
| Totally dependent for transfers | 1.693 (1.269–2.258) ^a)^ |
| **Nursing home residence (Ref. No)** | 1.464 (1.063–2.015) ^a)^ |
| **Hospitalization history (Ref. No) within 6-months before dialysis** | - |
| **Unplanned dialysis (Ref. planned)** | 1.487 (1.073–2.060) ^a)^ |
| **Blood urea nitrogen (per 1 mg/dL)** | - |
| **Creatinine (per 1 mg/dL)** | 0.902 (0.859–0.946) ^a)^ |
| **Albumin (per 1 g/dL)** | 0.528 (0.438–0.638) ^a)^ |
| **Inorganic phosphorus (per 1 mg/dL)** | - |
| **Number of patients per dialysis specialist at clinic level (per 1 patient)** | 1.013 (1.001–1.024) ^a)^ |

The multivariate model was adjusted for known significant factors and those showing statistical differences between the early and non-early mortality groups. The following parameters were used: age, sex, body mass index, hypertension, congestive heart failure, cardiac arrhythmia, peripheral vascular disease, active malignancy, severe behavioral disorder, mobility status, nursing home residence, hospitalization history within 6 months before dialysis, unplanned dialysis, blood urea nitrogen, creatinine, albumin, inorganic phosphorus levels, and number of dialysis specialists at clinic level. After excluding patients with missing values, 1,805 (97.0%) participants were included in the multivariate model.

^a)^ *p*-value <0.05.

CI, confidence interval; HR, hazard ratio; Ref., reference

**Supplementary Table S3. Multivariate Cox proportional hazards regression model analysis for early mortality of the number of patients per dialysis specialist at hospital level**

|  | **Multivariate HR (95% CI)** |
| --- | --- |
| **Age (per 1 years)** | 1.054 (1.034–1.074) ^a)^ |
| **Female (Ref. male)** | 0.823 (0.653–1.037) |
| **Body mass index (per 1 kg/m^2^)** | - |
| **Hypertension (Ref. No)** | - |
| **Congestive heart failure (Ref. No)** | - |
| **Cardiac arrhythmia (Ref. No)** | - |
| **Peripheral vascular disease (Ref. No)** | 0.532 (0.290–0.977) ^a)^ |
| **Active malignancy (Ref. No)** | 1.925 (1.213–3.056) ^a)^ |
| **Severe behavioral disorder (Ref. No)** | - |
| **Mobility** |  |
| Walking without help | Ref. |
| Needing assistance for transfers | 1.221 (0.924–1.614) |
| Totally dependent for transfers | 1.840 (1.380–2.454) ^a)^ |
| **Nursing home residence (Ref. No)** | 1.418 (1.031–1.950) ^a)^ |
| **Hospitalization history (Ref. No) within 6-months before dialysis** | - |
| **Unplanned dialysis (Ref. planned)** | 1.542 (1.115–2.133) ^a)^ |
| **Blood urea nitrogen (per 1 mg/dL)** | - |
| **Creatinine (per 1 mg/dL)** | 0.896 (0.853–0.941) ^a)^ |
| **Albumin (per 1 g/dL)** | 0.532 (0.440–0.642) ^a)^ |
| **Inorganic phosphorus (per 1 mg/dL)** | - |
| **Number of patients per dialysis specialist at hospital level (per 1 patient)** | 1.009 (1.002–1.015) ^a)^ |

The multivariate model was adjusted for known significant factors and those showing statistical differences between the early and non-early mortality groups. The following parameters were used: age, sex, body mass index, hypertension, congestive heart failure, cardiac arrhythmia, peripheral vascular disease, active malignancy, severe behavioral disorder, mobility status, nursing home residence, hospitalization history within 6 months before dialysis, unplanned dialysis, blood urea nitrogen, creatinine, albumin, inorganic phosphorus levels, and number of dialysis specialists at hospital level. After excluding patients with missing values, 1,805 (97.0%) participants were included in the multivariate model.

^a)^ *p*-value <0.05.

CI, confidence interval; HR, hazard ratio; Ref., reference

**Supplementary Table S4. Multivariate Cox proportional hazards regression model analysis for early mortality of the number of patients per dialysis specialist at general hospital level**

|  | **Multivariate HR (95% CI)** |
| --- | --- |
| **Age (per 1 years)** | 1.052 (1.032–1.072) ^a)^ |
| **Female (Ref. male)** | - |
| **Body mass index (per 1 kg/m^2^)** | - |
| **Hypertension (Ref. No)** | - |
| **Congestive heart failure (Ref. No)** | - |
| **Cardiac arrhythmia (Ref. No)** | - |
| **Peripheral vascular disease (Ref. No)** | 0.521 (0.284–0.954) ^a)^ |
| **Active malignancy (Ref. No)** | 2.133 (1.339–3.397) ^a)^ |
| **Severe behavioral disorder (Ref. No)** | - |
| **Mobility** |  |
| Walking without help | Ref. |
| Needing assistance for transfers | 1.308 (0.994–1.720) |
| Totally dependent for transfers | 2.034 (1.534–2.697) ^a)^ |
| **Nursing home residence (Ref. No)** | - |
| **Hospitalization history (Ref. No) within 6-months before dialysis** | - |
| **Unplanned dialysis (Ref. planned)** | 1.617 (1.168–2.239) ^a)^ |
| **Blood urea nitrogen (per 1 mg/dL)** | - |
| **Creatinine (per 1 mg/dL)** | 0.898 (0.856–0.942) ^a)^ |
| **Albumin (per 1 g/dL)** | 0.536 (0.444–0.647) ^a)^ |
| **Inorganic phosphorus (per 1 mg/dL)** | - |
| **Number of patients per dialysis specialist at general hospital level (per 1 patient)** | 1.025 (1.012–1.039) ^a)^ |

The multivariate model was adjusted for known significant factors and those showing statistical differences between the early and non-early mortality groups. The following parameters were used: age, sex, body mass index, hypertension, congestive heart failure, cardiac arrhythmia, peripheral vascular disease, active malignancy, severe behavioral disorder, mobility status, nursing home residence, hospitalization history within 6 months before dialysis, unplanned dialysis, blood urea nitrogen, creatinine, albumin, inorganic phosphorus levels, and number of dialysis specialists at general hospital level. After excluding patients with missing values, 1,805 (97.0%) participants were included in the multivariate model.

^a)^ *p*-value <0.05.

CI, confidence interval; HR, hazard ratio; Ref., reference

**Supplementary Table S5. Comparison of baseline characteristics between early mortality and non-early mortality groups in the propensity score matched cohort**

|  | Early mortality group  (n=321) | Non-early mortality group  (n=642) | *p*-value |
| --- | --- | --- | --- |
| **Age (years)** | 79.7±5.9 | 79.4±5.5 | 0.375 |
| **Male (%)** | 177 (55.1) | 336 (52.3) | 0.411 |
| **Body mass index (kg/m^2^)** | 22.6±4.0 | 22.7±3.8 | 0.805 |
| **Comorbidities** |  |  |  |
| Diabetes mellitus (%) | 182 (56.7) | 367 (57.2) | 0.890 |
| Hypertension (%) | 270 (84.1) | 569 (88.6) | 0.049 |
| Ischemic heart disease (%) | 75 (23.4) | 145 (22.6) | 0.786 |
| Congestive heart failure (%) | 65 (20.2) | 114 (17.8) | 0.349 |
| Cardiac arrhythmia (%) | 55 (17.1) | 84 (13.1) | 0.092 |
| Cerebrovascular disease (%) | 59 (18.4) | 125 (19.5) | 0.685 |
| Peripheral vascular disease (%) | 12 (3.7) | 41 (6.4) | 0.089 |
| Active malignancy (%) | 20 (6.2) | 22 (3.4) | 0.045 |
| Severe behavioral disorder (%) | 57 (17.8) | 89 (13.9) | 0.112 |
| Liver cirrhosis (%) | 14 (4.4) | 24 (3.7) | 0.640 |
| **Mobility** |  |  | <0.001 |
| Walking without help (%) | 134 (41.7) | 331 (51.6) |  |
| Needing assistance for transfer (%) | 93 (29.0) | 201 (31.3) |  |
| Totally dependent for transfer (%) | 94 (29.3) | 110 (17.1) |  |
| **Nursing home residence (%)** | 52 (16.2) | 66 (10.3) | 0.008 |
| **Hospitalization history within 6 months before dialysis (%)** | 141 (43.9) | 245 (38.2) | 0.085 |
| **Unplanned dialysis (%)** | 276 (86.0) | 512 (79.8) | 0.018 |
| **Laboratory findings** |  |  |  |
| Hemoglobin (g/dL) | 9.2±1.6 | 9.1±1.6 | 0.653 |
| Blood urea nitrogen (mg/dL) | 73±37 | 74±34 | 0.668 |
| Creatinine (mg/dL) | 5.3±2.8 | 5.6±2.5 | 0.148 |
| Albumin (g/dL) | 3.1±0.6 | 3.1±0.6 | 0.657 |
| Calcium (mg/dL) | 8.2±0.9 | 8.1±0.9 | 0.014 |
| Inorganic phosphorus (mg/dL) | 4.7±1.9 | 4.7±2.3 | 0.823 |
| **Number of patients per dialysis specialist** | 56.3±7.1 | 54.6±7.3 | 0.001 |
| **Proportions of centers that did not meet the recommended number of dialysis specialists (%)** | 50.2±11.4 | 47.7±11.9 | 0.002 |

Continuous and categorical variables are presented as mean±standard deviation and as number (%), respectively.

**Supplementary Table S6. Analysis results of previous early mortality prediction models in the present study cohort**

|  | **AuROC value** | **95% CI** |
| --- | --- | --- |
| **REIN score^a)^ [17]** | 0.681 | 0.649–0.713 |
| **Santos score^b)^ [18]** | 0.621 | 0.588–0.653 |
| **Thamer score^c)^ [19]** | 0.675 | 0.643–0.707 |

^a)^ The REIN score was composed of age, sex, congestive heart failure, peripheral vascular disease, cardiac arrhythmia, active malignancy, severe behavioral disorder, mobility status, and serum albumin. ^b)^ Santos et al.’s score was composed of age, ischemic heart disease, cerebrovascular disease, serum albumin, and prior nephrologist's care, but prior nephrologist's care scores were omitted in the present study as they were not collected.
^c)^ Thamer et al.’s score was composed of age, serum albumin, mobility status, nursing home residence, malignancy, congestive heart failure, and hospitalization history within 6-months before dialysis.
AuROC, area under the receiver operating curve; CI, confidence interval; REIN, renal epidemiology and information network cohort
